# Supplementary material for: Structure and function of the soil microbiome underlying N2O emissions from global wetlands
Source: Nat Commun. 2022 Mar 17;13:1430. doi: 10.1038/s41467-022-29161-3 (PMC8931052; doi:10.1038/s41467-022-29161-3)
Supplement: Supplementary file 1 — Supplementary Information [file 41467_2022_29161_MOESM1_ESM.pdf]

Supplementary Information for Bahram et al. "Structure and function of the soil microbiome underlying N_2O emissions from global wetlands"

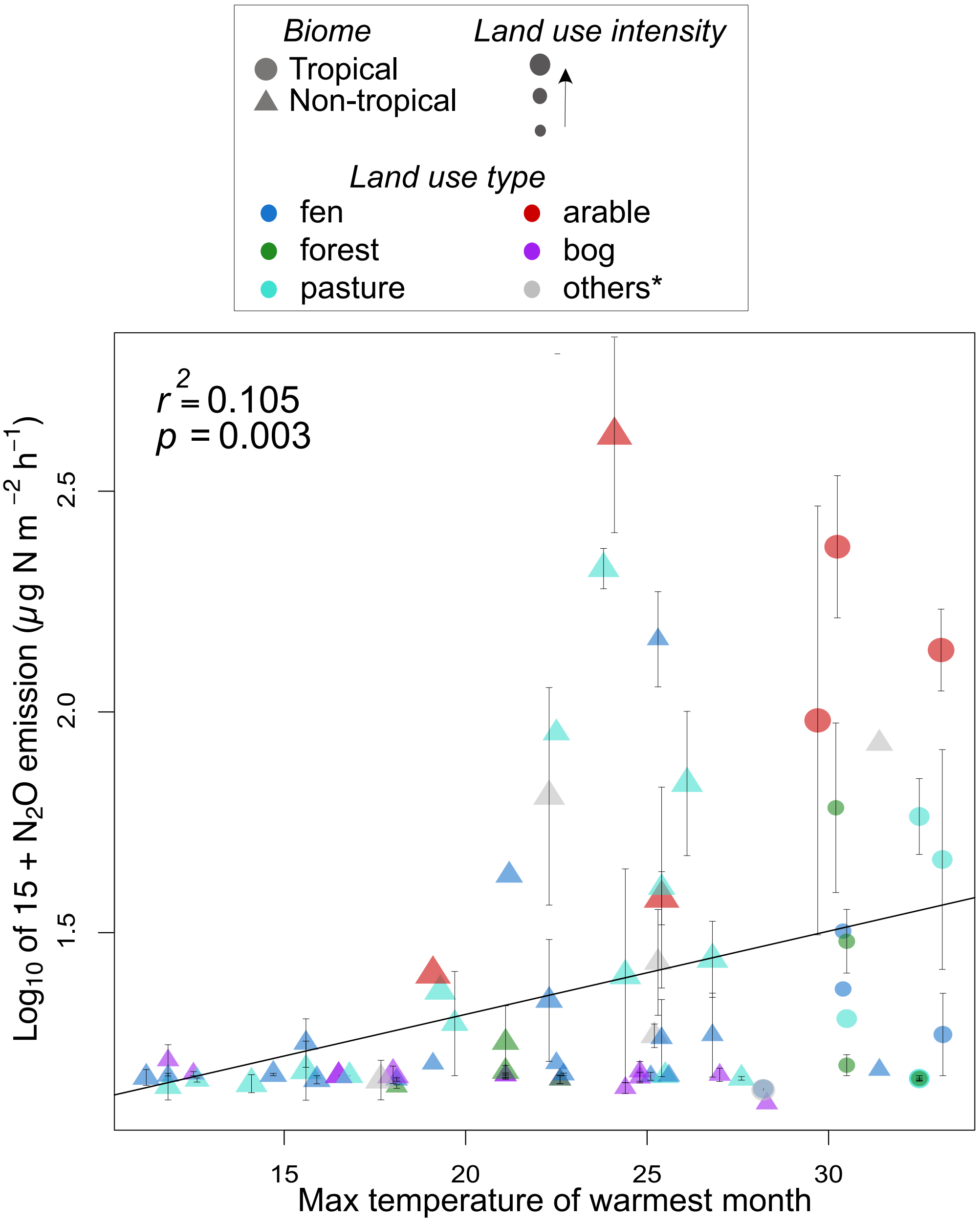

Supplementary Fig. 1. **Relationship between N_2O emission and maximum temperature of the warmest month.** Symbol color corresponds to land-use type, symbol shape corresponds to biome type, and symbol size corresponds to the level of land-use intensity, as indicated in the legend. Error bars represent the standard errors (SE) of the means (n=74 independent sites). * Land-use types with few representative sites (<=3) are shown in the same color for better visualization, including peat extraction, recreation, and hay field.

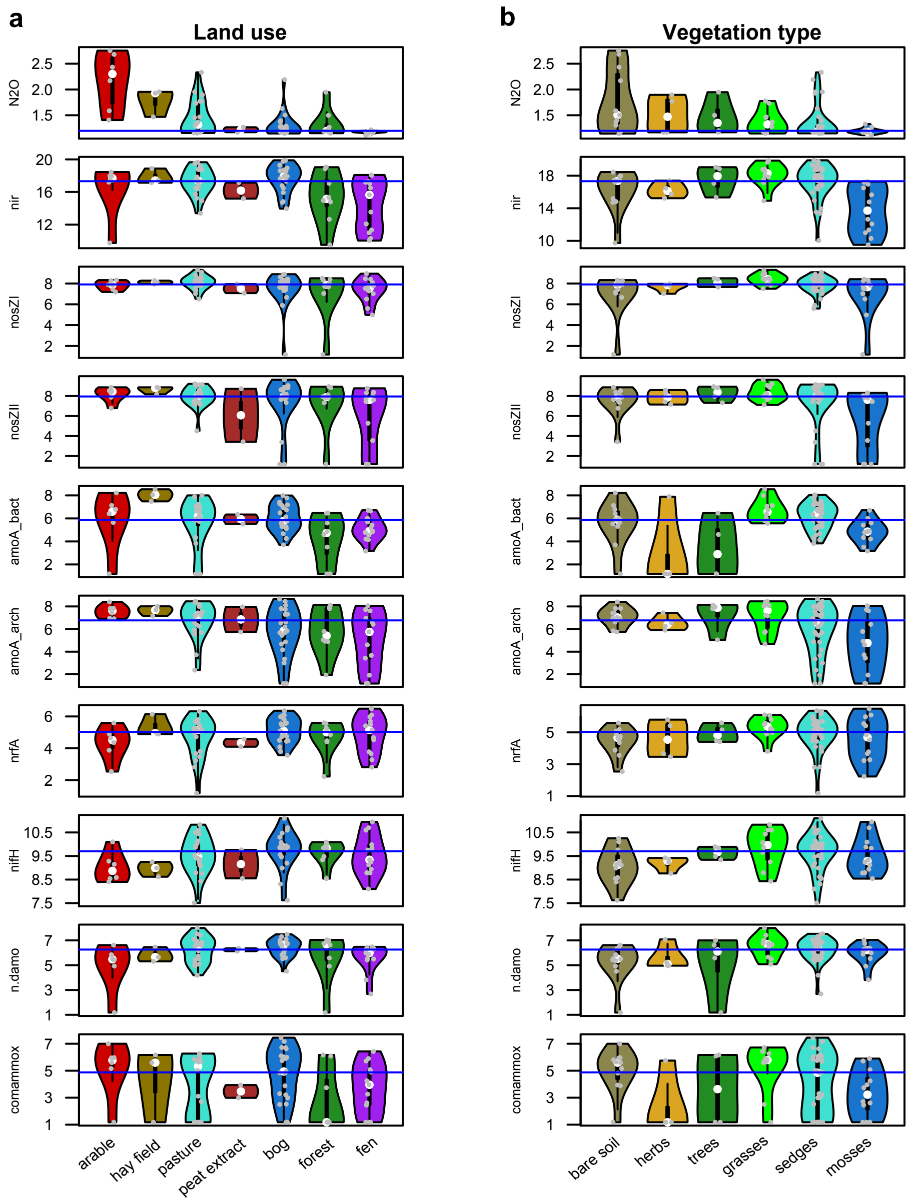

Supplementary Fig. 2. Distribution of N_2O emission and the abundance of nitrogen cycling genes across land-use (a) and vegetation types (b). The blue horizontal lines indicate the median across the whole dataset. Gene abundances were quantified based on qPCR. All values on y-axis are presented in log scale (log10 +15). Violin plots represent 25th–75th percentile of the data distribution with whiskers at 1.5 × the interquartile range and the middle line representing median. Each point represents a site (n=74 independent sites). The shape of the violin plot represents the probability density of the data at different values.

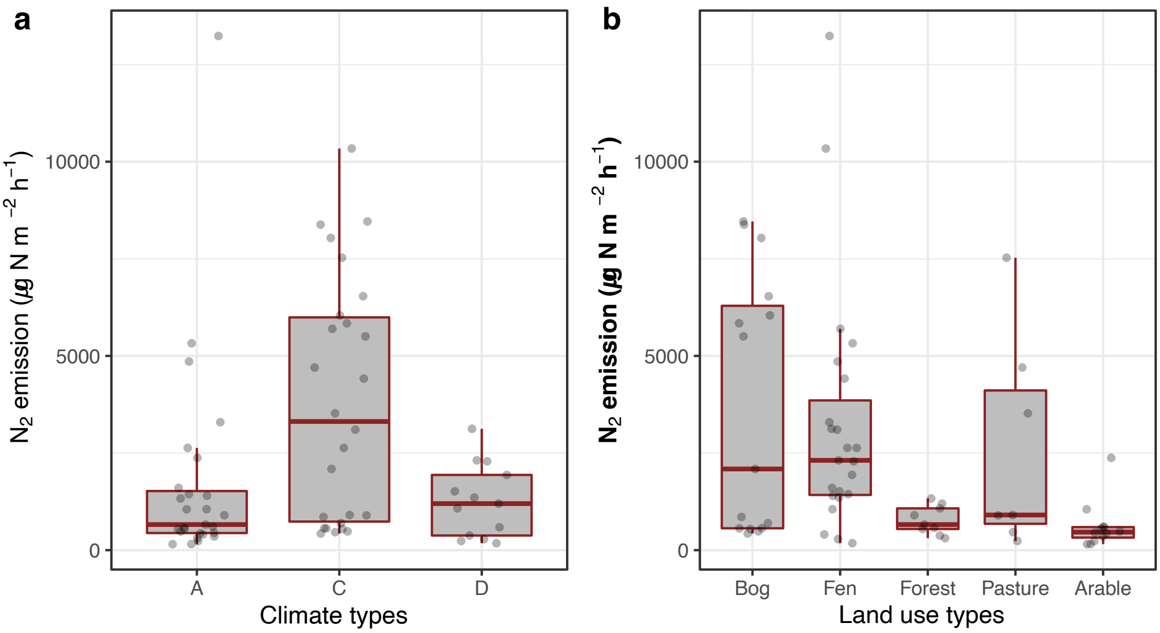

Supplementary Fig. 3. The potential N_2 emissions from soil related to Köppen climate types (a, see Methods) and land use types (b). Boxes represent 25th–75th percentile of the data distribution with whiskers at 1.5 × the interquartile range and the middle line representing median. Each point represents a site (n=66 independent sites). N_2 emissions were negatively correlated with land-use intensity (Spearman r=-0.254, p= 0.040).

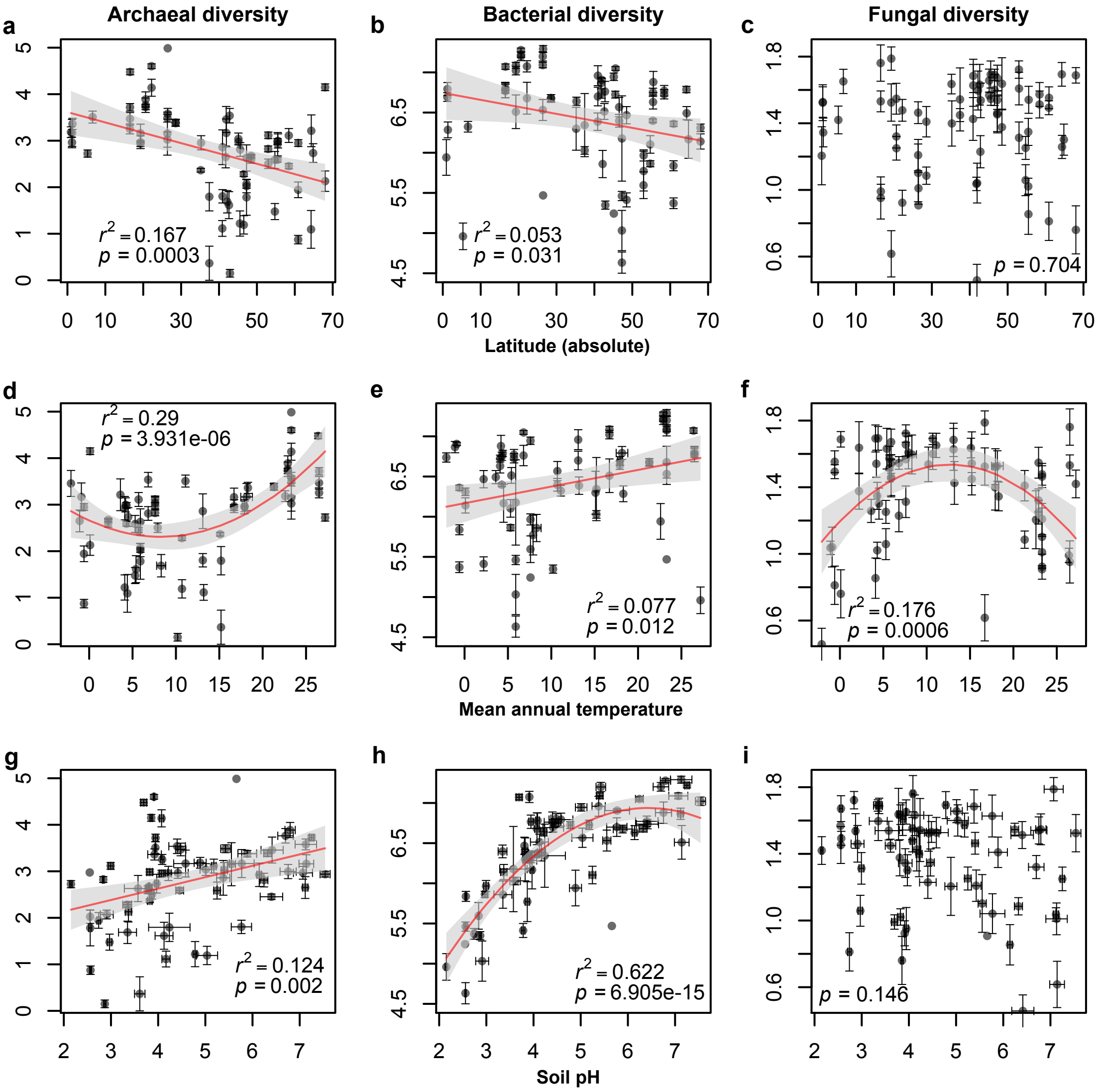

Supplementary Fig. 4. Latitudinal gradients of archaeal (left panels), bacterial (central panels) and fungal (right panels) diversity across the global wetland soils. Diversity is calculated as the Shannon diversity index on the 16S rRNA gene metabarcoding dataset (Illumina) for archaea and bacteria as well as the ITS metabarcoding dataset (PacBio) for fungi. The inset numbers represent r^2adj from linear or second order polynomial regressions chosen based on the lowest AIC score for each relationship. Error bars represent the standard errors (SE) of the means (n=74 independent sites). The statistical test used was two-sided.

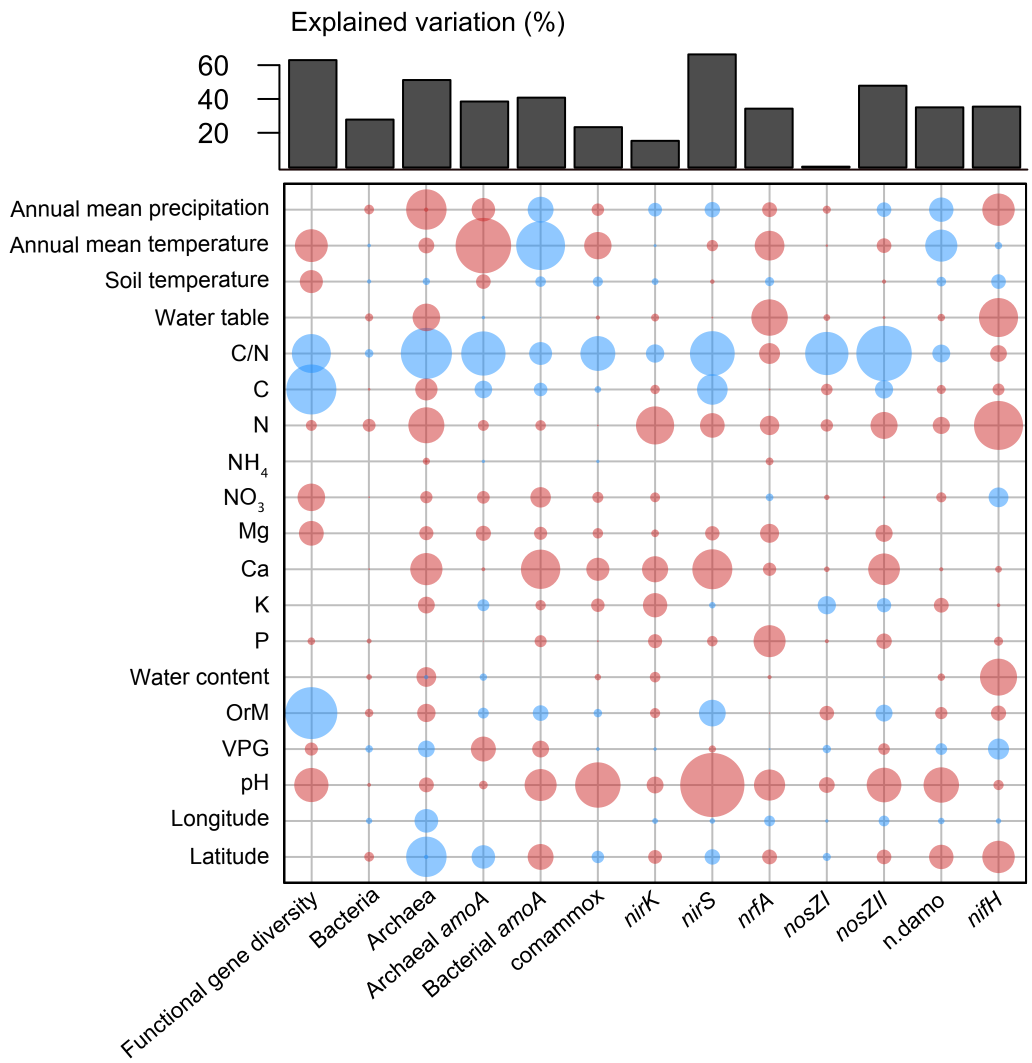

Supplementary Fig. 5. The main determinants of the diversity and abundance of key microbial groups involved in N_2O dynamics across global wetland soils. The size of circles corresponds to the variable importance based on Random Forest models (% of mean decrease accuracy estimated based on out-of-bag-CV); blue and red depict negative and positive Spearman correlations, respectively (n=74 independent sites). Functional gene diversity is the Shannon diversity index calculated based on the absolute abundance of key N-cycle genes involved in N_2O dynamics quantified by qPCR (see Supplementary Table 9). Other response variables represent absolute abundances quantified by qPCR. The abbreviations are OrM (organic matter), MAT (mean annual temperature), MAP (mean annual precipitation), pH (soil pH), C/N (soil carbon to nitrogen ratio), VPG (Von Post grade of decomposition).

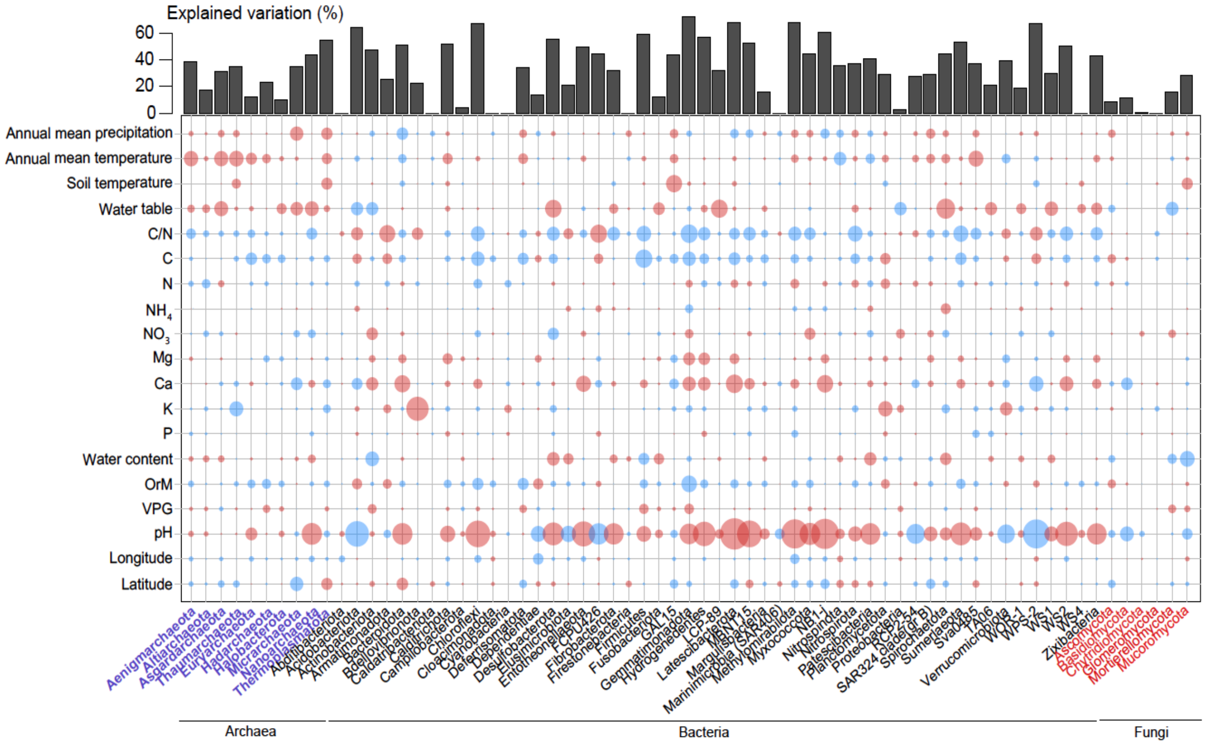

Supplementary Fig. 6. Environmental predictors of major archaeal, bacterial, and fungal phyla across the global wetland soils. These data are based on the relative abundance of SSU rRNA genes as revealed by metabarcoding (Illumina for prokaryotes and PacBio for fungi). The size of circles corresponds to the partial importance based on Random Forest models (variability% of mean decrease in accuracy estimated based on out-of-bag-CV). Blue and red colors depict negative and positive Spearman correlations, respectively (n=74 independent sites). The abbreviations are OrM (organic matter), MAT (mean annual temperature), MAP (mean annual precipitation), pH (soil pH), C/N (soil carbon to nitrogen ratio) and VPG (Von Post grade of decomposition).

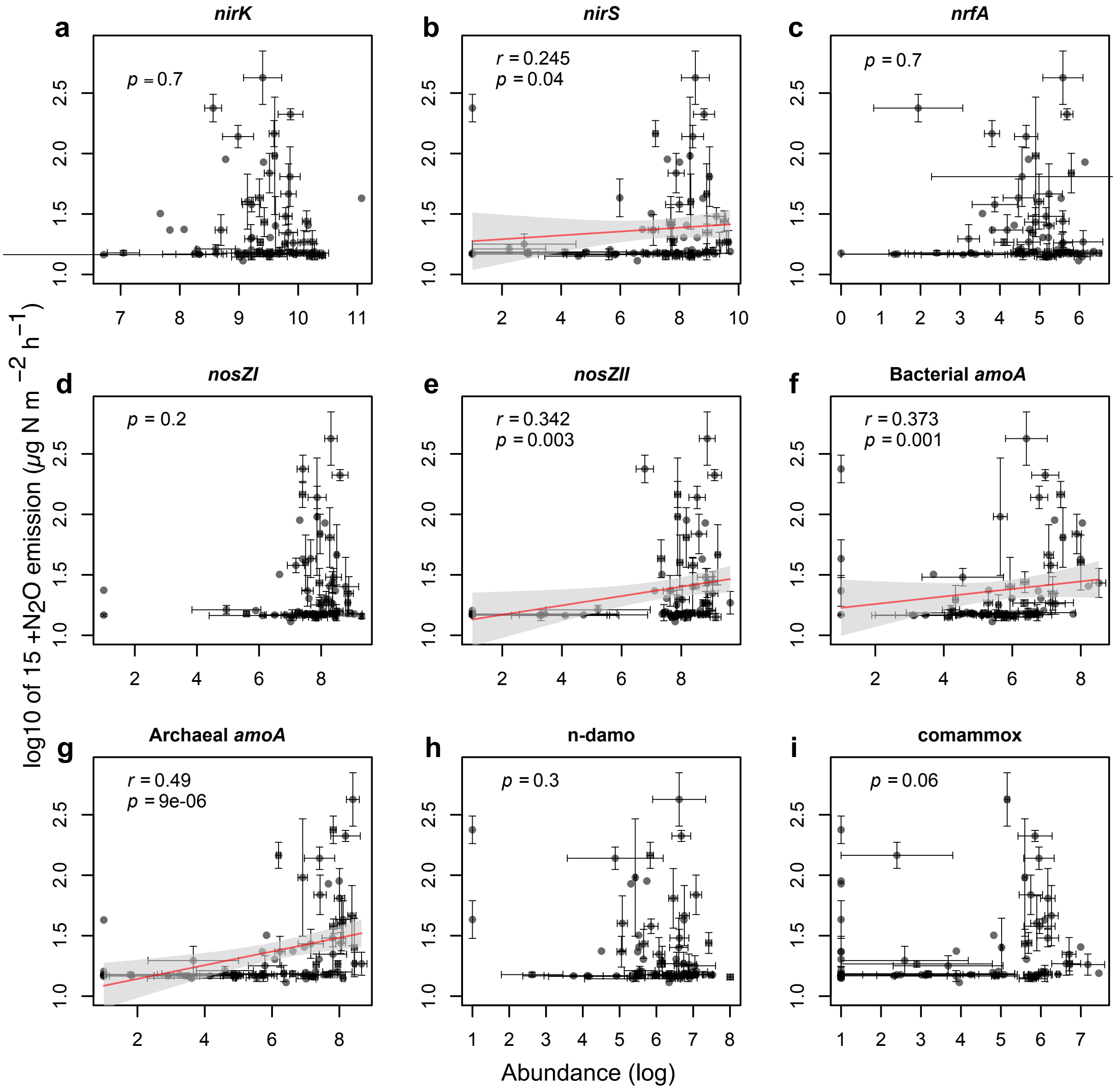

Supplementary Fig. 7. Relationship between N_2O emission and abundance of major N cycling genes involved in N_2O dynamics. The abundance of prokaryotes and their functional genes were quantified using qPCR. Error bars represent the standard errors (SE) of the means (n=74 independent sites). The statistical test used was two-sided.

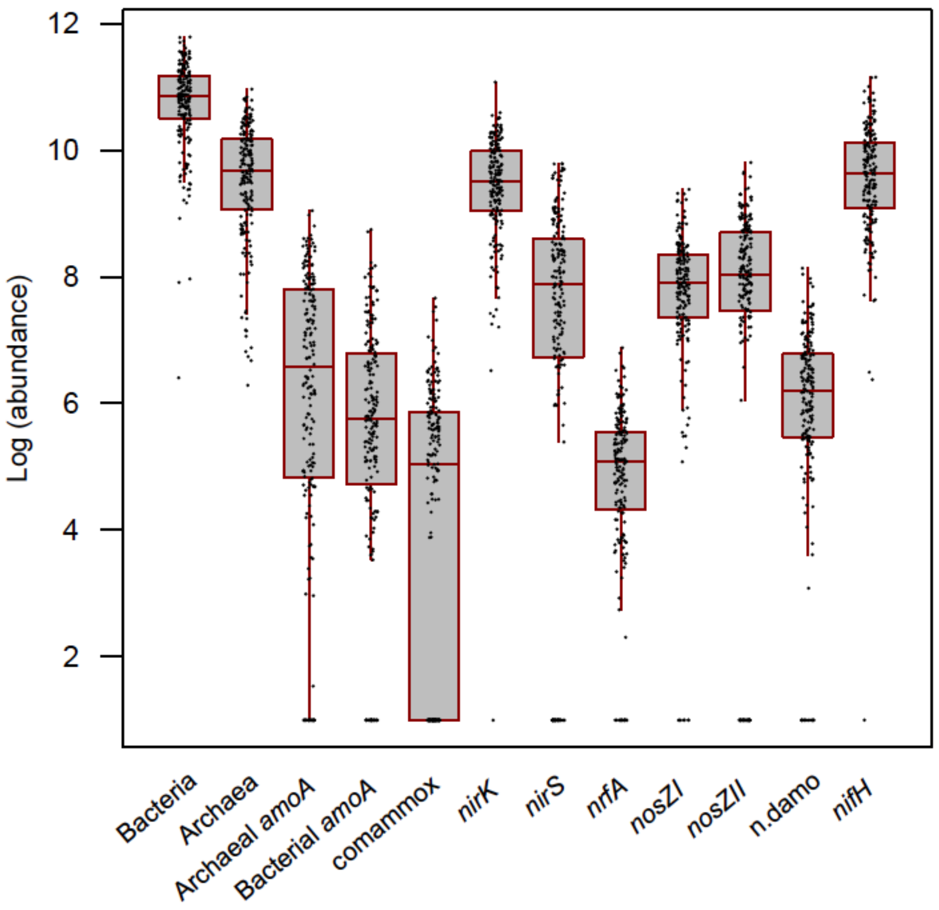

Supplementary Fig. 8. Abundance of nitrogen (N) cycling genes in global wetland soils. Box and whisker plots showing the absolute abundances (determined by qPCR) of the main N cycling genes across the global wetland soil samples. Boxes represent 25th–75th percentile of the data distribution with whiskers at 1.5 × the interquartile range and the middle line representing median. Each point represents a site (n=74 independent sites).

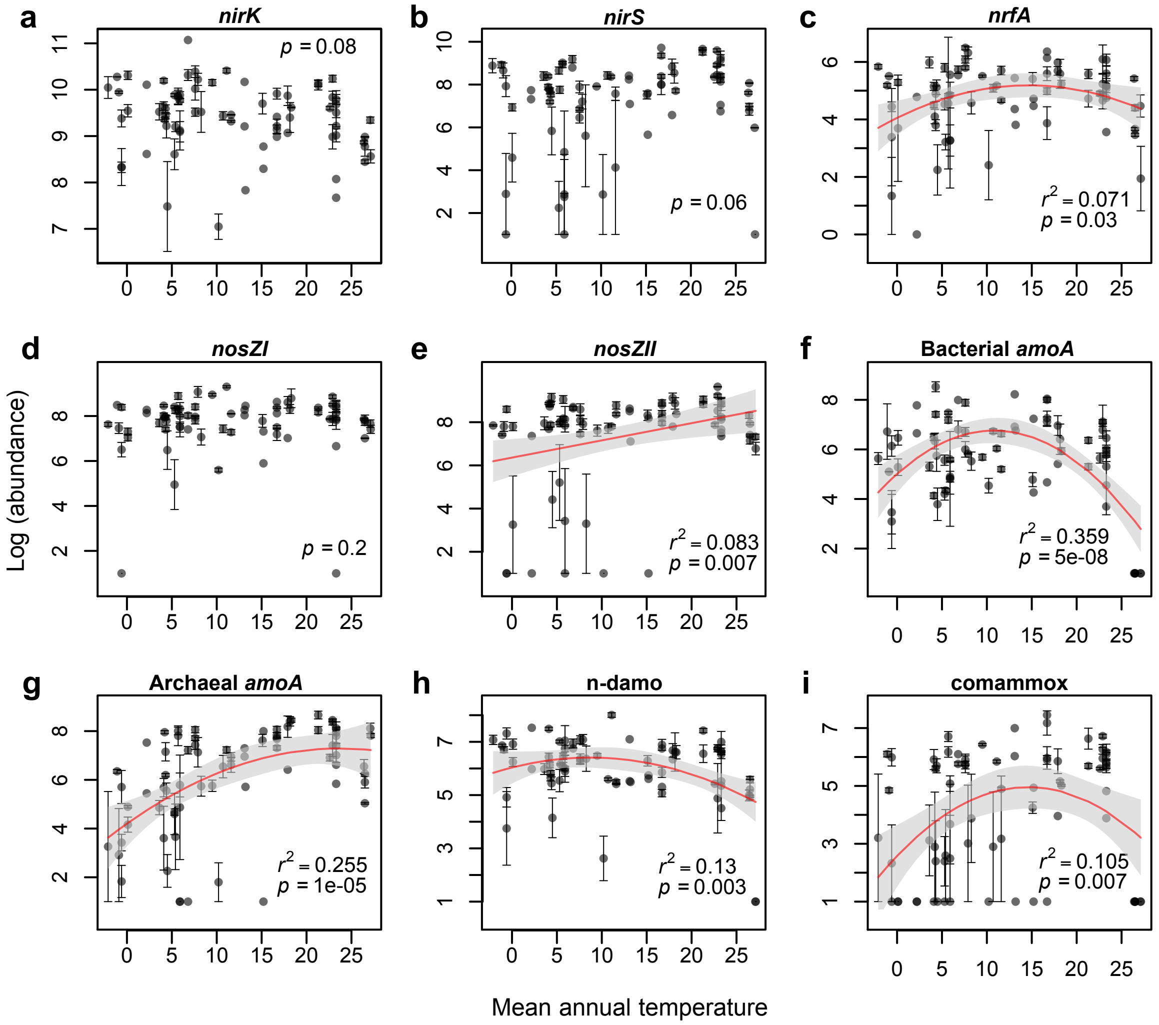

Supplementary Fig. 9. Relationship between temperature and the abundance of main genes involved in N_2O dynamics. The scatterplots showing the relationship between the abundance of N cycle genes (as determined by qPCR) and mean annual temperature. The inset numbers represent r^2adj from linear or second order polynomial regressions chosen based on the lowest AIC score for each relationship. Error bars represent the standard errors (SE) of the means (n=74 independent sites). The statistical test used was two-sided.

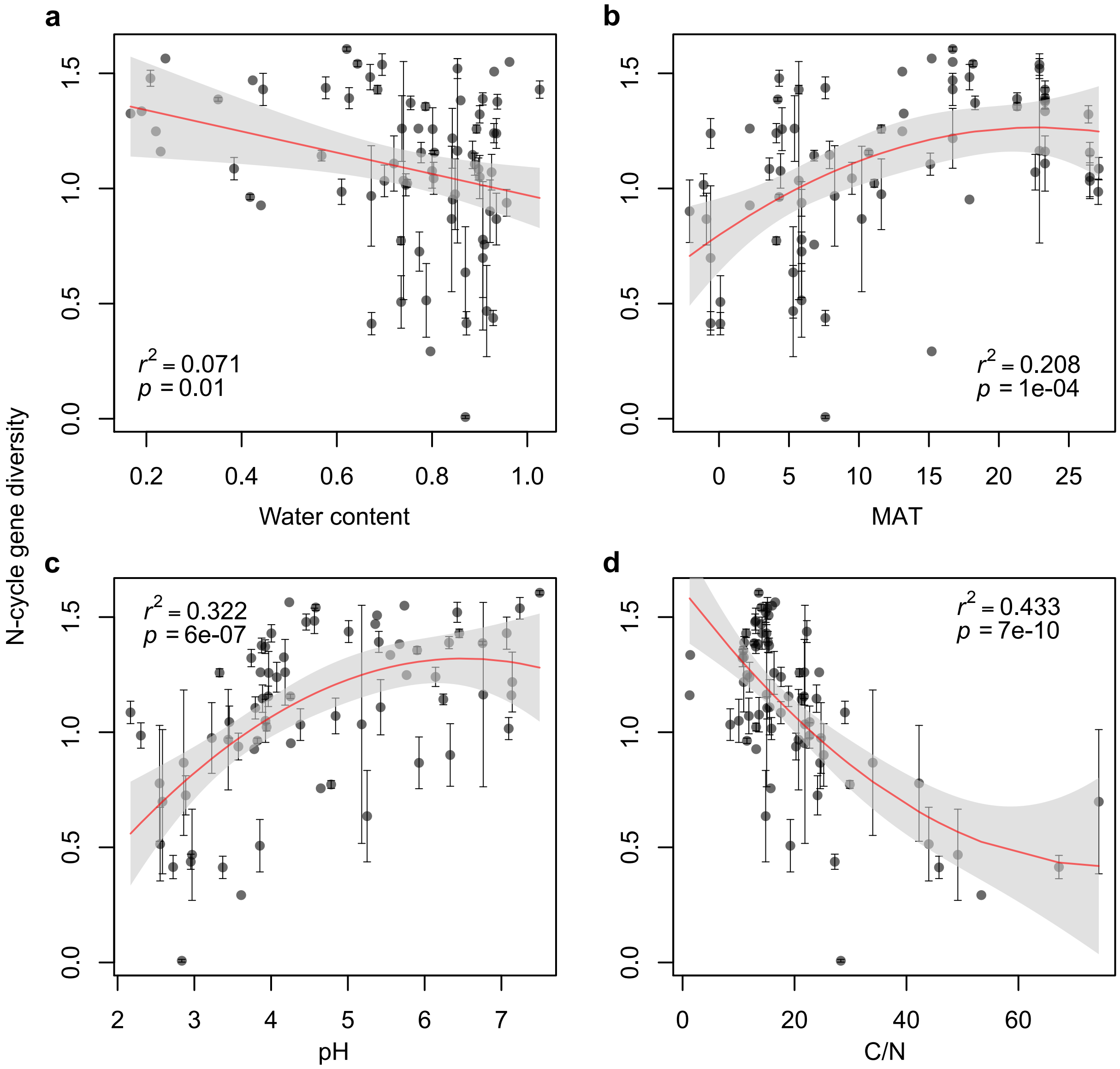

Supplementary Fig. 10. Relationships between the diversity of functional N-cycle genes and environmental characteristics, including (a) soil water content, (b) mean annual temperature, (c) soil pH and (d) soil carbon to nitrogen ratio (C/N). N-cycle gene diversity is the Shannon diversity index calculated based on the absolute abundance of key N-cycle genes quantified by qPCR. The inset numbers represent r^2adj from linear or second order polynomial regressions chosen based on the lowest AIC score for each relationship. Error bars represent the standard errors (SE) of the means (n=74 independent sites). The statistical test used was two-sided. The abbreviations are MAT (mean annual temperature), pH (soil pH), C/N (soil carbon to nitrogen ratio).

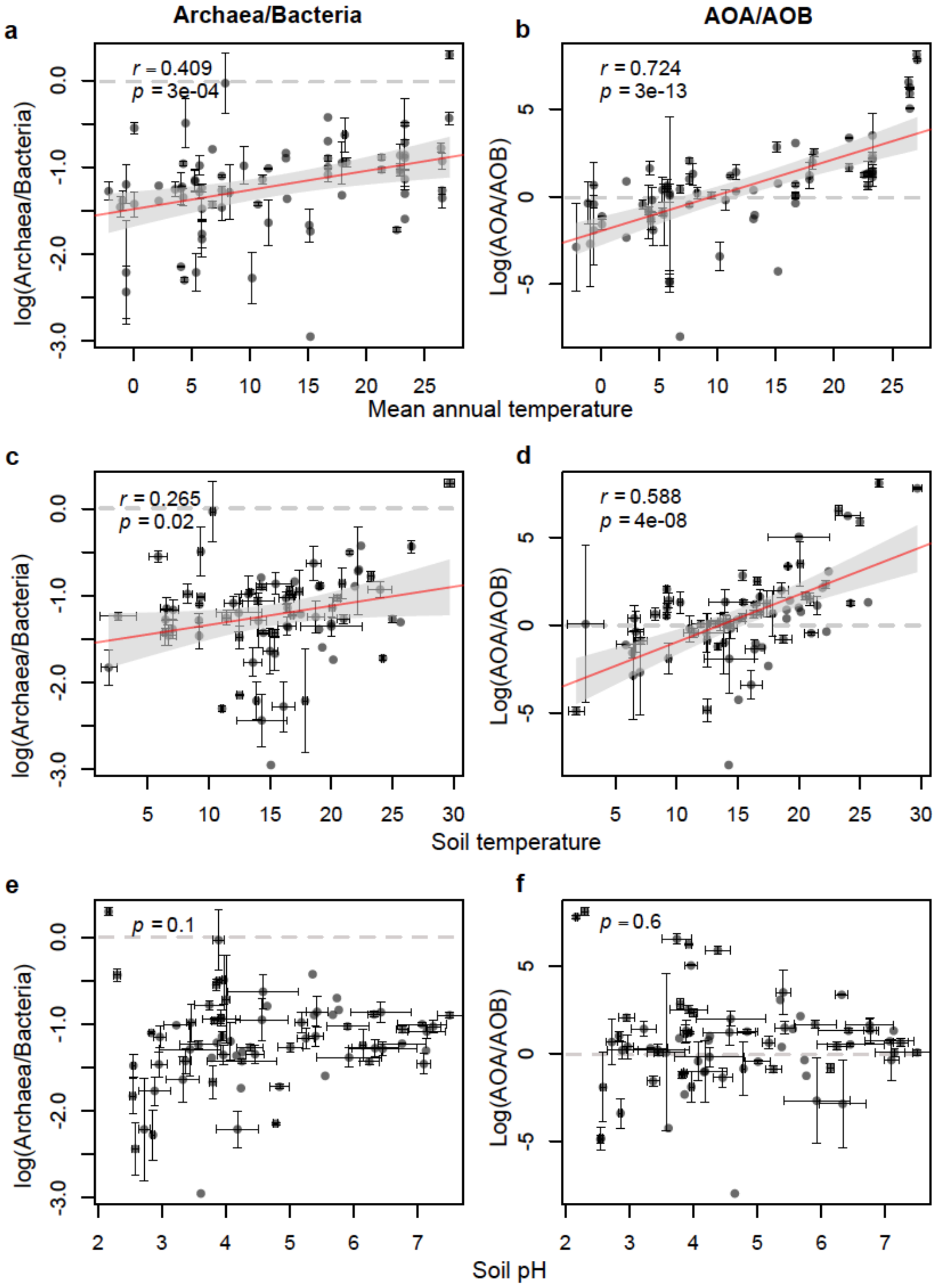

Supplementary Fig. 11. **Niche separation of AOA and AOB across the global climate gradient independently of soil pH.** The scatterplots showing the relationship between log-ratio of archaeal to bacterial 16S rRNA gene (**a, c, e**) and *amoA* (**b, d, f**) gene (quantified based on qPCR) and mean annual temperature (**a, b**), soil temperature at 20 cm depth (**c, d**) and soil pH (**e, f**). Error bars represent the standard errors (SE) of the means (n=74 independent sites). The statistical test used was two-sided.
