# Supplementary material for: Structure and function of the soil microbiome underlying N2O emissions from global wetlands
Source: Nat Commun. 2022 Mar 17;13:1430. doi: 10.1038/s41467-022-29161-3 (PMC8931052; doi:10.1038/s41467-022-29161-3)
Supplement: Supplementary file 3 — Description of Additional Supplementary Files [file 41467_2022_29161_MOESM3_ESM.pdf]

Description of Additional Supplementary Files

Supplementary Data 1. Samples used in this study.

Supplementary Data 2. Correlations between N_2O emissions and the relative abundance of all genera uncovered by metagenomes.

Supplementary Data 3. List of archaeal OTUs showing strongest correlations to N2O fluxes.

Supplementary Data 4. Correlation between N_2O fluxes and Archaeal OGs across global wetland soils.

Supplementary Data 5. Comparison between the functional gene profiles (MetaCyc and KEGG modules) of N2O taxa (determined using PacBio metabarcoding) that showed strongest positive and negative correlations with N2O production.

Supplementary Data 6. List of genomes that contain genes related to ammonia oxidation from a total of 91 archaeal and 116 bacterial genomes.

Supplementary Data 7. Analysis of MetaCyc nitrogen metabolism pathways in available Archaeal genomes in JGI as off 15-jul-2020.

Supplementary Data 8. Characteristics of qPCR primer pairs and programs used.
